# Changes in crypt cell DNA content during experimental colonic carcinogenesis.

**DOI:** 10.1038/bjc.1986.133

**Published:** 1986-06

**Authors:** J. Matthews, T. Cooke

## Abstract

Changes in colonic crypt cell DNA content have been monitored during experimental carcinogenesis. Colonic tumours were induced in Wistar rats using 12 consecutive subcutaneous injections of azoxymethane at a dose of 10 mg kg-1. Ten rats were killed at each of 10, 15, 20 and 25 weeks after the initial injection. On sacrifice the descending colon, plus any polyps or tumours with their adjacent mucosa, was removed, fixed and processed to paraffin wax. Sections were stained for DNA by the Feulgen reaction. Using an integrating microdensitometer the DNA content of the proliferative and functional cells was measured and expressed as a percentage of the stem cell DNA content. As carcinogenesis progressed there was an increase in the mean amount of DNA in the proliferative and functional cells in the distal colon although the tissue was histologically normal. The transitional mucosa adjacent to tumours showed the same increases as the 25 week distal colon. In the adenomas, there was a further increase in the DNA content of the functional cells. These results are probably a reflection of the increase in the number of dividing cells in the higher positions of the colonic crypts during carcinogenesis.


*Br. J. Cancer* (1986), **53**, 787–791

### Changes in crypt cell DNA content during experimental colonic carcinogenesis

J. Matthews & T. Cooke

*Department of Surgery, Charing Cross and Westminster Medical School, London W6 8RP, UK.*

**Summary** Changes in colonic crypt cell DNA content have been monitored during experimental carcinogenesis. Colonic tumours were induced in Wistar rats using 12 consecutive subcutaneous injections of azoxymethane at a dose of $10\,mg\,kg^{-1}$. Ten rats were killed at each of 10, 15, 20 and 25 weeks after the initial injection. On sacrifice the descending colon, plus any polyps or tumours with their adjacent mucosa, was removed, fixed and processed to paraffin wax. Sections were stained for DNA by the Feulgen reaction. Using an integrating microdensitometer the DNA content of the proliferative and functional cells was measured and expressed as a percentage of the stem cell DNA content. As carcinogenesis progressed there was an increase in the mean amount of DNA in the proliferative and functional cells in the distal colon although the tissue was histologically normal. The transitional mucosa adjacent to tumours showed the same increases as the 25 week distal colon. In the adenomas, there was a further increase in the DNA content of the functional cells. These results are probably a reflection of the increase in the number of dividing cells in the higher positions of the colonic crypts during carcinogenesis.

Prognosis for patients who present with Dukes' A large bowel tumours is good with 5 year survival as high as 100% (Gill & Morris, 1978). Detection of very early tumours or even pre-neoplasia would lead to an improved survival for individual patients and might have some impact in reducing morbidity for the disease as a whole. Screening may be particularly beneficial in individuals at high risk of developing colonic carcinoma, for example those with multiple adenomas, chronic inflammatory bowel diseases or after 'curative' surgery for large bowel cancer (Lipkin, 1984).

The use of conventional biopsy and histology is of limited value in interpreting precancerous changes in the bowel, for example, the presence of inflammatory cells may interfere with the diagnosis of dysplasia in ulcerative colitis (Morson, 1972). Conventional histology will confirm advanced and even some early neoplastic changes, but it is a less reliable method of detecting subtle preneoplastic changes especially at the nuclear level. It has been suggested that an increase in the sialomucin content of colonic mucosa is an indicator of premalignancy (Filipe, 1975) although hypersecretion of sialomucin has also been demonstrated in non-neoplastic diseases of the colon (Litensey & Riddel, 1981; Franzin *et al.*, 1983). These alterations in the mucin patterns may be interpreted as reactive and a specific phenomenon to ischaemic and/or inflammatory stimuli rather than indicators of premalignant change.

Colonic crypts have an orderly kinetic organisation, changes in which can be detected using stathmokinetic or thymidine labelling techniques (Cooke *et al.*, 1984; Wright & Allison, 1984). An alteration of the kinetic organisation of the crypts is seen in patients with polyposis coli and ulcerative colitis with an increase in the size of the proliferative compartment (Bleiberg *et al.*, 1972; Eastwood & Trier, 1973; Lipkin, 1977). Transitional mucosa adjacent to tumours also shows these changes, with mitoses being seen at higher levels of the crypts than in normal epithelium. The amount of DNA in an individual cell reflects its stage in the cell cycle so proliferative changes in colonic mucosa may be reflected by an alteration in the mean DNA content of the crypt cells. The Feulgen technique stains DNA stoichiometrically and the amount of stain in individual cells can be measured by cytophotometry. It is possible therefore that this technique may be used to detect kinetic changes in the colonic mucosa.

In this study, we have evaluated these methods by using a model of colorectal neoplasia induced by the cycasin derivative azoxymethane which produces colonic tumours histologically similar to human colonic carcinoma (Sunter, 1980). The DNA contents of cells in three different areas of the colonic crypts has been investigated in the precancerous colonic mucosa in an attempt to determine whether pre-neoplasia can be recognised.

### Materials and methods

### *Animals*

Male cob Wistar rats with an initial weight of 250 g were housed 4 to a cage and were fed on a

Correspondence: J. Matthews
Received 22 November 1985; and in revised form 27 January 1986.

© The Macmillan Press Ltd., 1986

standard laboratory diet with free access to water. Carcinogenesis was induced by 12 weekly s.c. injections of azoxymethane $10 \mathrm{mg\,kg^{-1}}$ body weight in 4 groups of 10 rats. The experiment was designed to monitor premalignant changes in the colon before the appearance of frank carcinoma in the majority of animals. The first group was sacrificed immediately after the eleventh injection and subsequent groups were killed at 15, 20 and 25 weeks after the initial injection. Controls were given 12 weekly injections of saline and were sacrificed at 25 weeks.

Animals were killed under deep ether anaesthesia and their colons were removed, opened longitudinally and washed with normal saline. A 1 cm length of distal colon from each animal, taken from a standard site (25% the length of the colon from the anal margin) and any colonic tumours together with their adjacent mucosa were fixed in 10% neutral buffered formol saline.

### *Morphology*

All of the fixed tissue was routinely processed and embedded in paraffin wax. Two serial $5\,\mu m$ sections of both the tumours and standard site colon were cut. One section from each piece of tissue was stained with haematoxylin and eosin for morphological assessment and the second section was stained for DNA.

### *Histochemistry*

Sections were dewaxed and rehydrated through graded alcohols, dipped briefly in 1N hydrochloric acid at room temperature and transferred to 5N hydrochloric acid, again at room temperature for 45 min. The sections were then immersed, in the dark, in Schiff's reagent for 1 h and washed in 3 changes of bisulphite water (2:1 vol:vol, 0.5% potassium metabisulphite to 1N hydrochloric acid). After rinsing in distilled water, the sections were dehydrated, cleared in inhibisol and mounted in DPX.

### *DNA measurement*

The colonic crypts in the mucosa from the standard site, and those adjacent to tumours and from polyps were divided into 3 zones. Zone 1 included cells in the bottom 3 positions of the crypt, zone 2 cells between positions 15 and 20 in the central portion of the crypt and zone 3 cells in the top 3 positions of the crypt. The amount of DNA, measured in units of relative absorbance, in one cell from each zone in 10 adjacent countable crypts was measured in each specimen. Cells were selected on their suitability to be counted, i.e. if there was no overlap with other nuclei and if it was obvious (by size) that the nuclei had not been sectioned through a pole. Measurements were made by means of a Vickers M85 scanning and integrating microdensitometer at 550 nm with a ×40 objective and a scanning spot size of $0.5\,\mu m$ in the plane of the specimen. A mask which had a diameter of $6\,\mu m$ was used for all measurements.

### *Reproducibility*

To test the reproducibility of the technique, 4 serial $5\,\mu m$ sections of normal rat colon were stained for DNA by the Feulgen technique, and on each section the DNA content of 10 cells from each zone, as previously described, was measured.

### *Statistics*

As precise thickness could not be guaranteed for all of the sections used and because there was batch variability in the staining, the mean relative absorbance of cells from zones 2 and 3 in each animal were expressed as a percentage of the mean relative absorbance of cells in zone 1. A paired $t$ test was used to compare the cells from the 3 zones and a Student's $t$ test used to compare the different weeks of carcinogenesis.

### Results

### *Reproducibility*

The mean relative absorbance for each zone in each section is given in Table I. There were no significant differences between the values obtained within the zones. In 3 of the sections the cells in zone 1 had a higher but insignificant mean relative absorbance than those in zone 2 and in zone 3. In the fourth section the mean relative absorbance for zones 1 and 2 were the same although the value for zone 3 was reduced.

**Table I** Mean relative absorbance (±s.e.m.) of cells from zones 1, 2 and 3 in four serial sections of normal rat colon

| *Section* | *Zone 1* | *Zone 2* | *Zone 3* |
|---|---|---|---|
| 1 | 12.0±2.68 | 10.1±1.09 | 9.9±1.03 |
| 2 | 11.6±1.00 | 10.9±1.72 | 10.2±1.03 |
| 3 | 12.2±3.25 | 10.7±1.55 | 9.4±1.41 |
| 4 | 11.5±0.99 | 11.5±2.64 | 9.2±1.24 |

### *Changes in DNA during carcinogenesis*

The changes in cellular DNA content during carcinogenesis in zones 2 and 3 relative to zone 1 are shown in Table II. In control animals the mean relative absorbance decreased from 12.6 in zone 1 to 10.7 in zone 2, a mean reduction of 15.3±2.31%

**Table II** Mean reduction in DNA content of cells from zones 2 and 3 as a percentage of the DNA content of cells in zone 1 (±s.e.m.)

| | *Zone 2* | *Zone 3* |
|---|---|---|
| Control | 15.3±2.31 | 23.7±3.12 |
| 10 weeks | 6.9±2.52 | 17.7±3.24 |
| 15 weeks | 4.6±1.22 | 16.5±1.81 |
| 20 weeks | 3.1±2.16 | 9.1±2.87 |
| 25 weeks | [a]2.5±3.63 | 13.4±2.52 |
| Adjacent to tumours | 0.3±3.18 | 16.4±2.50 |
| Adenomas | [a]5.3±2.50 | [a]4.5±2.40 |

[a]Represents an increase in DNA content compared to zone 1.

($P<0.05$). There was a further decrease in mean absorbance to 9.5 in zone 3, a reduction of 23.7 ±3.12% compared to zone 1 ($P<0.001$). The difference between zones 2 and 3 was statistically significant ($P<0.01$). These differences between the DNA contents of the 3 zones were maintained up to week 15 of carcinogenesis. By week 20 there was no significant difference between zones 1 and 2, although in zone 3 there was a mean reduction of 9.1±2.8% compared with zone 1 ($P<0.01$).

The percentage of DNA in the cells from zone 2 compared to zone 1 increased throughout carcinogenesis from 85% in the control animals to 103% in the 25 week animals (Figure 1). By week 10, the percentage was already significantly greater than in the controls ($P<0.05$). DNA in the cells in zone 3 compared to zone 1 also increased through carcinogenesis from 76% in the control groups to 87% in the 25 week group.

**Figure 1** Graph showing the DNA content of cells from zones 2 and 3 as a percentage of that from zone 1. Asterisks represent significant differences from the control values (*$P<0.05$, **$P<0.01$, ***$P<0.001$).

Despite these alterations in DNA contents during carcinogenesis, all of the tissue taken from the standard site was histologically normal.

### *Mucosa adjacent to tumours*

Colonic tumours were found in 7 of the rats at 25 weeks of carcinogenesis. Of these 3 were adenocarcinoma and the rest were adenomas with areas of severe dysplasia (polyps). The mucosa adjacent to the tumours showed the same results as the mucosa taken from the standard site in the 25 week group (Table II). There was no significant difference between the mean DNA contents of the cells from zones 1 and 2 but there was a mean reduction of 16.4±2.50% in zone 3 compared to zone 1 ($P<0.001$).

In the polyps, there was no significant difference between the DNA contents of the cells from any of the 3 zones.

### Discussion

In this study we have investigated cellular DNA content at various levels in the colonic crypts throughout carcinogenesis and have found an increase in the DNA content of cells in the upper regions of the colonic crypts as carcinogenesis progresses.

### *Technique*

The use of sections for measuring DNA content of cells may present problems due to inconsistent section thickness and staining variation. These problems are overcome by using each section as its own control i.e. by comparing the DNA contents of zones 2 and 3 to that of zone 1 in each individual section. Different proportions of nuclei within individual sections may pose a second problem although Fordham *et al.* (1985), when comparing the results of flow cytometric analysis of whole nuclei from disaggregated thick sections of prostatic tumours with the results of microdensitometry of Feulgen stained 5 μm sections of the same tumours, found that the two techniques produced very similar results.

When comparing the results from the 4 serial sections of normal colon, the spread of relative absorbances of nuclei in zone 1 was greater than that of zone 2 which was greater than that of zone 3. This spread was reflected in the standard errors of the results and is due to the presence of more dividing cells in the lower regions of the crypts than in the upper regions.

In this study, the number of nuclei measured for each animal was small so no meaningful interpretation of results from individual animals could be made, but by looking at 10 animals from each group an overall conclusion could be drawn. If this technique were to be used in man, many more cells would have to be measured so that significant differences between the mean cellular DNA contents of the zones could be obtained in individual patients. This is probably not practical in a screening situation as it would prove too labour intensive.

*Cell kinetics*

In a normal colonic crypt, the cells at the base of the crypt, the stem cells, are actively dividing, although the cell cycle time of these cells is long so few mitotic figures are actually seen in this area (Rijke *et al.*, 1979; Sunter *et al.*, 1979). As the cells migrate up the crypts into the proliferative zone, cell cycle times reduce and the mitotic index rises (Al-Dewachi *et al.*, 1974; Al-Dewachi *et al.*, 1979; Sunter *et al.*, 1979). This is the major zone of crypt cell production above which proliferation gradually tails off to zero (around cell position 22) due to the steady reduction in the proportion of cycling cells. In the maturation zone, cell division is no longer taking place so no mitotic figures or cells in S phase or in G2 will be present in the upper parts of the colonic crypts.

In the control animals, between cell positions 15 and 20, only half the number of cells in the distal colon are dividing compared with cells between positions 1 and 4 which explains the decrease in the mean amount of DNA per cell in this region compared to that in the stem cell zone. Similarly, because no cells are in division at the top of the crypts, these cells will contain less DNA than the mean value for cells both at the base and in the middle of the crypts.

Using the same animal model, we have found that during carcinogenesis there is an increase in the crypt cell production rate in the descending colon (Cooke *et al.*, 1984). This leads to an increase in the size of the proliferative compartment and to an increase in the mean cellular DNA content in the middle of the crypts. The cellular DNA content may increase due to aneuploidy occurring during carcinogenesis but this technique cannot distinguish aneuploid cells from cells in the S or G2 phase of the cell cycle.

In adenomas, which in this model are believed to be premalignant lesions (Cooke *et al.*, 1984), the mean cellular DNA levels are the same throughout the length of the crypts indicating that all of these cells have the same reproductive capacity. It is also possible that some of these polyps are composed of clones of aneuploid cells.

In man, cell proliferation is also confined to the lower two thirds of the colonic crypts, but in patients with polyposis coli, a disease known to be associated with a greatly increased incidence of colonic cancer, dividing cells can be detected by tritiated thymidine labelling in the upper third of morphologically normal crypts (Deschner & Lipkin, 1975). Although this technique is probably not feasible in man, we have been able to observe proliferative changes associated with premalignancy. However, staining of single cells from the upper regions of the crypt by the Feulgen technique would be possible and by measuring the DNA content on an automated system to detect proliferative changes, it would provide a useful screening system for patients at risk of developing colonic tumours.

**References**

AL-DEWACHI, H.S., WRIGHT, N.A., APPLETON, D.R. & WATSON, A.J. (1974). The cell cycle time in the rat jejunal mucosa. *Cell Tissue Kinet.*, **7**, 587.

AL-DEWACHI, H.S., APPLETON, D.R., WATSON, A.J. & WRIGHT, N.A. (1979). Variation in the cell cycle time in the crypts of Lieberkuhn of the mouse. *Virchow's Arch* (*Cell Pathol.*), **31**, 37.

BLEIBERG, H., MAINGUET, P. & GALAND, P. (1972). Cell renewal in familial polyposis. Comparison between polyps and adjacent healthy mucosa. *Gastroenterology*, **63**, 240.

COOKE, T., KIRKHAM, N., STAINTHORP, D.H., INMAN, C., GOETING, N. & TAYLOR, I. (1984). Detection of early neoplastic changes in experimentally induced colorectal cancer using scanning electron microscopy and cell kinetic studies. *Gut*, **25**, 748.

DESCHNER, E.E. & LIPKIN, M. (1975). Proliferative patterns in colonic mucosa in familial polypsis. *Cancer*, **35**, 413.

EASTWOOD, G.L. & TRIER, J.S. (1973). Epithelial cell renewal in cultured rectal biopsies in ulcerative colitis. *Gastroenterology*, **64**, 383.

FILIPE, M.I. (1975). Mucous secretion in rat colonic mucosa during carcinogenesis induced by dimethylhydrazine. A morphological and histochemical study. *Br. J. Cancer*, **32**, 60.

FORDHAM, M.V.P., MATTHEWS, J., WILLIAMS, G. & COOKE, T. (1985). DNA content of prostate tumours. *Eur. J. Surg. Oncol.*, **11**, 316.

FRANZIN, G., GRIGIONI, W.F., DINA, R., SCARPA, A. & ZAMBONI, G. (1983). Mucin secretion and morphological changes of the mucosa in non-neoplastic disease of the colon. *Histopathology*, **7**, 707.

GILL, P.G. & MORRIS, P.J. (1978). The survival of patients with colorectal cancer treated in a regional hospital. *Br. J. Surg.*, **65**, 17.

LIPKIN, M. (1977). Growth kinetics of normal and premalignant gastrointestinal epithelium. In *Growth Kinetics and Biochemical Regulation of Normal and Malignant Cells*, p. 562. Williams and Wilkins: Baltimore.

LIPKIN, M. (1984). The identification of high risk populations. *Scan. J. Gastroenterol.*, **19** (Suppl. 104), 91.

LITENSKY, C.M. & RIDDEL, R.H. (1981). Patterns of mucin secretion in neoplastic and non-neoplastic diseases of the colon. *Human Pathol.*, **12**, 923.

MORSON, B.C. (1972). Rectal biopsy in inflammatory bowel disease. *N. Engl. J. Med.*, **287**, 1337.

RIJKE, R.P., PLAISIER, H.M. & LANGENDOEN, N.J. (1979). Epithelial cell kinetics in the descending colon of the rat. *Virchow's Arch* (*Cell Pathol.*), **30**, 85.

SUNTER, J.P., WATSON, A.J., WRIGHT, N.A. & APPLETON, D.R. (1979). Cell proliferation at different sites along the length of the rat colon. *Virchow's Arch* (*Cell Pathol.*), **32**, 75.

SUNTER, J.P. (1980). Experimental carcinogenesis and cancer in the rodent gut. In *Cell Proliferation in the Gastro-intestinal Tract*, Appleton, D.R., Sunter, J.P. & Watson, A.J. (eds) p. 255. Pitman Medical: London.

WRIGHT, N. & ALISON, M. (1984). The biology of epithelial cell populations. p. 605. Clarendon Press: Oxford.


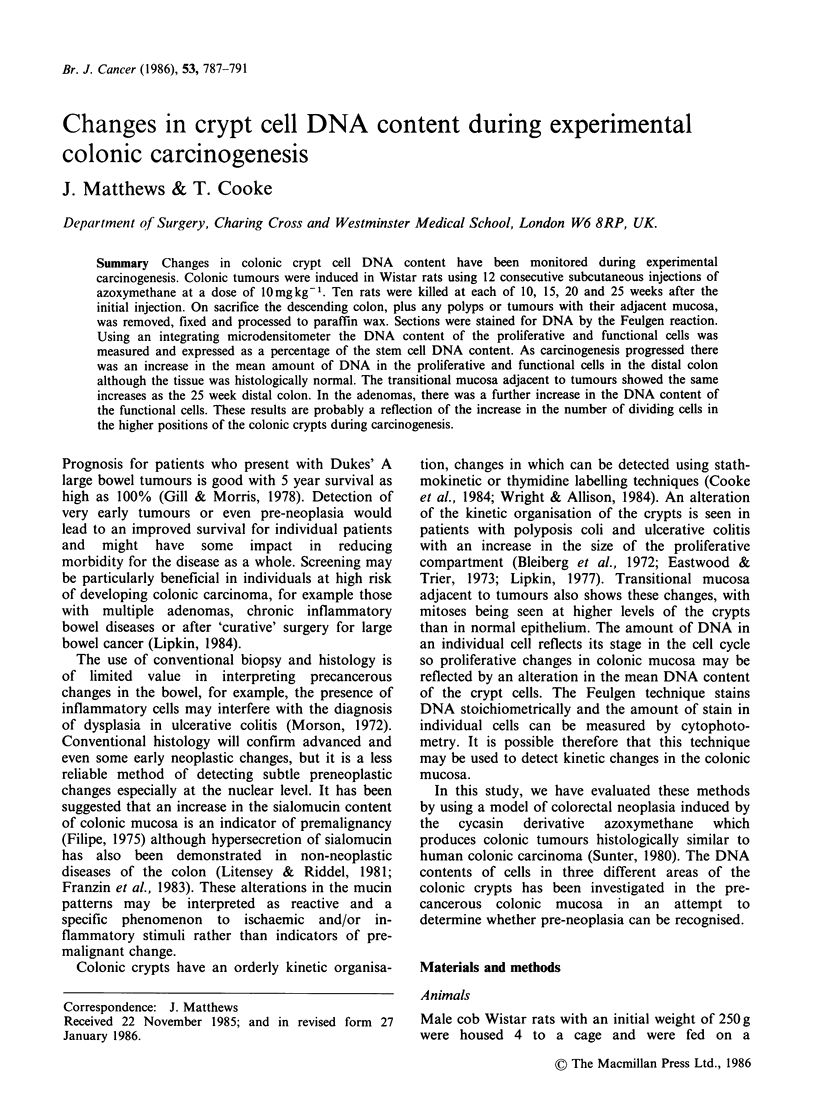

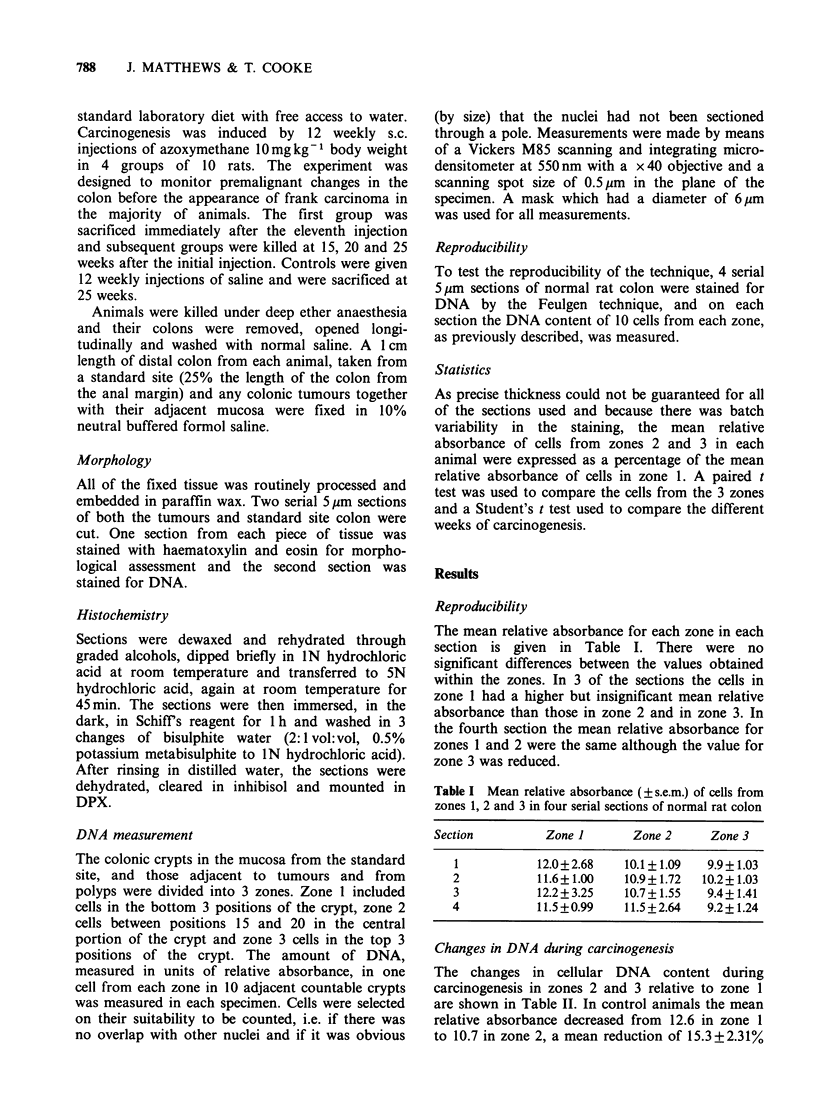

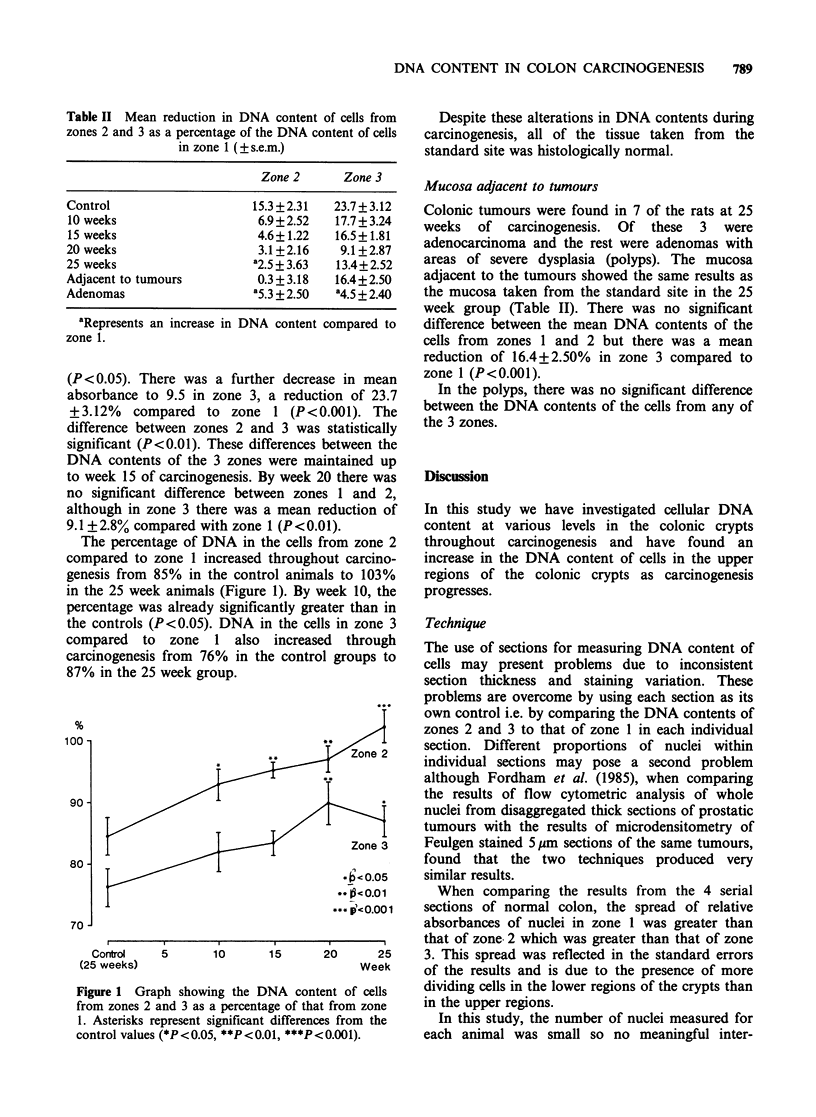

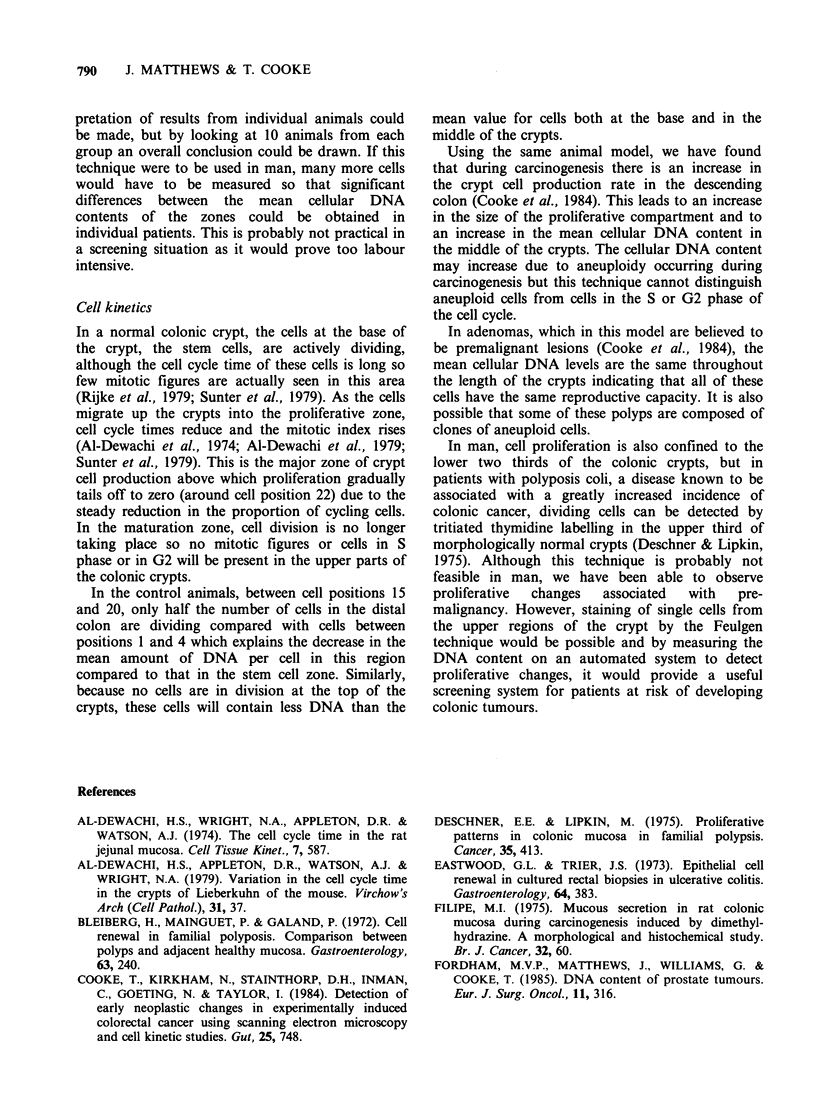

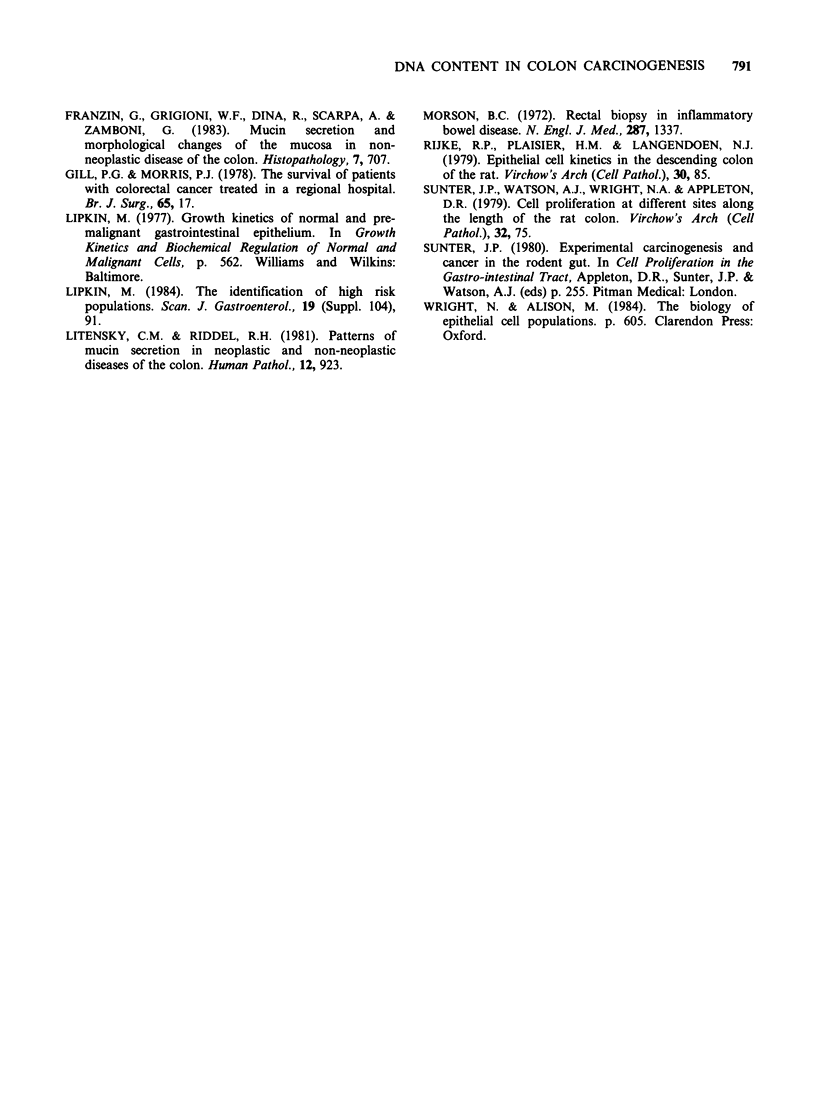


## References

[OCR_00442] Al-Dewachi H. S., Wright N. A., Appleton D. R., Watson A. J. (1974). The cell cycle time in the rat jejunal mucosa.. Cell Tissue Kinet.

[OCR_00453] Bleiberg H., Mainguet P., Galand P. (1972). Cell renewal in familial polyposis: comparison between polyps and adjacent healthy mucosa.. Gastroenterology.

[OCR_00459] Cooke T., Kirkham N., Stainthorp D. H., Inman C., Goeting N., Taylor I. (1984). Detection of early neoplastic changes in experimentally induced colorectal cancer using scanning electron microscopy and cell kinetic studies.. Gut.

[OCR_00466] Deschner E. E., Lipkin M. (1975). Proliferative patterns in colonic mucosa in familial polyposis.. Cancer.

[OCR_00471] Eastwood G. L., Trier J. S. (1973). Epithelial cell renewal in cultured rectal biopsies in ulcerative colitis.. Gastroenterology.

[OCR_00476] Filipe M. I. (1975). Mucous secretion in rat colonic mucosa during carcinogenesis induced by dimethylhydrazine. A morphological and histochemical study.. Br J Cancer.

[OCR_00489] Franzin G., Grigioni W. F., Dina R., Scarpa A., Zamboni G. (1983). Mucin secretion and morphological changes of the mucosa in non-neoplastic diseases of the colon.. Histopathology.

[OCR_00495] Gill P. G., Morris P. J. (1978). The survival of patients with colorectal cancer treated in a regional hospital.. Br J Surg.

[OCR_00507] Lipkin M. (1984). The identification of high risk populations.. Scand J Gastroenterol Suppl.

[OCR_00512] Listinsky C. M., Riddell R. H. (1981). Patterns of mucin secretion in neoplastic and non-neoplastic diseases of the colon.. Hum Pathol.

[OCR_00517] Morson B. C. (1972). Rectal biopsy in inflammatory bowel disease.. N Engl J Med.

